# Re-examination of perinatal mental health policy frameworks for women signalling distress on the Edinburgh Postnatal Depression Scale (EPDS) completed during their antenatal booking-in consultation: a call for population health intervention

**DOI:** 10.1186/s12884-019-2378-4

**Published:** 2019-07-02

**Authors:** Sarah Khanlari, Bryanne Barnett AM, Felix Akpojene Ogbo, John Eastwood

**Affiliations:** 1 Department of Community Paediatrics, Sydney Local Health District, Croydon Community Health Centre, 24 Liverpool Street, Croydon, NSW 2132 Australia; 2 School of Medicine and Public Health, University of Newcastle, Callaghan Campus, University Drive, Callaghan, NSW 2308 Australia; 3 Sydney Institute for Women Children and their Families, Sydney Local Health District, Camperdown, NSW 2050 Australia; 4 School of Psychiatry, Faculty of Medicine, University of New South Wales, Kensington Campus, Kensington, NSW 2052 Australia; 5 Translational Health Research Institute, School of Medicine, University of Western Sydney, Campbelltown Campus, Locked Bag 1797, Penrith, NSW 2571 Australia; 6 Ingham Institute for Applied Medical Research, 1 Campbell Street, Liverpool, NSW 2170 Australia; 7 School of Women’s and Children’s Health, Faculty of Medicine, The University of New South Wales, Kensington, Sydney, NSW 2052 Australia; 8 Menzies Centre for Health Policy, Charles Perkins Centre, School of Public Health, Sydney University, Sydney, NSW 2006 Australia

**Keywords:** Perinatal, Pregnancy, Distress, Depression, EPDS, Screening, Policy, Risk-stratification, Prevention

## Abstract

**Background:**

Globally, anxiety and depression are the most common complications of the perinatal period (conception to 1 year postpartum). It is now recognised that anxiety and depression are more commonly found antenatally than postnatally and represent the greatest risk factor for developing postnatal depression. Research in this space has focused on treatment of postnatal depression, with limited attention paid to preventative strategies for women signalling distress, who are subthreshold for diagnosable illness.

**Main text:**

The Edinburgh Postnatal Depression Scale (EPDS) was introduced in 1987 and has since been validated as a depression screening tool in the Australian and international setting. The EPDS has been embedded as a depression screening tool within a broader psychosocial assessment for women who receive their maternity care in the public system in Australia. Owing to perceived service restrictions, an EPDS score must reach a threshold of 13 or more to warrant specific assessment or intervention. Current policy frameworks focus on tertiary prevention models, and those women scoring 10 to 12, who could reasonably be considered as signalling distress or early signs of illness, are not currently offered intervention. The consequences of undetected or untreated perinatal mood or anxiety disorder (often co-morbid) include maternal psychological, social, occupational and physical dysfunction, and extend to deleterious infant and child life-course effects. This provides a strong justification to explore the role of preventative programs for women who are distressed. A range of low-resource, population-based interventions are available and effective. We explore the evidence for a selection of these programs. Further research is needed to decipher their effectiveness as a secondary prevention approach in women who are currently signalling distress during antenatal assessment.

**Conclusion:**

The burden of perinatal mood disorders, and their potential for prolonged impact, justify the exploration of preventatively-focused programs in women who signal distress during antenatal care.

## Background

Globally, mental health disorders, including substance use, account for 7.4% of disability-adjusted life years (DALYs), with anxiety and depressive disorders accounting for 55% of this burden [1]. By the year 2030, it is predicted that unipolar depression will be in the top three leading causes of disease burden, together with ischaemic heart disease and human immunodeficiency virus (HIV) [2]. Anxiety and depressive disorders are the most common complication of pregnancy and the first postnatal year, known as the perinatal period, affecting 10–15% of women [3–6], although this figure ranges from 4 to 64% and varies considerably between and within countries [7]. Trauma and suicide are the leading causes of death in the perinatal period in Australia, with the majority of cases having had psychiatric or substance use issues identified [8]. Suicide and accidental injury are classified together because it is understood that many deaths coded as ‘trauma’ were possibly intentional [9]. A review of maternal deaths in New South Wales Australia between 2000 and 2006 highlighted the violent means by which the majority of women suicide in the first postpartum year [8, 9], and is mirrored in the British context [10]. This represents an important contrast to the predominant methods of suicide used by females who are neither pregnant nor postpartum, though these trends are less striking in more recently studied populations [11].

The range of mental health disorders in the ante- and postnatal period is often subsumed under the umbrella label of ‘perinatal or postnatal depression’, with anxiety disorders receiving appropriate attention more recently [12]. We now also recognise, separately from the anxiety disorders, the trauma, stressor-related and personality disorders that can be diagnosed in the perinatal period, although they are often missed [13–15]. While there is little conjecture regarding the need for responsive and targeted approaches for women who are recognised as having a probable current mental illness, less resource has been allocated to elucidating preventative pathways. Many women go on to develop postnatal mental health disorders without any formal recognition of antenatal precursors, despite common risk factors being identifiable. Pregnant women experience distress along a spectrum and subclinical symptom levels contribute to pregnancy and delivery problems as well as the increased likelihood of further deterioration postpartum [16]. Accurate detection of these women in the antenatal period with a validated predictive tool holds potential promise for population-based interventions to be offered.

The aim of this review is to provide a contextual history to the use of the Edinburgh Postnatal Depression Scale (EPDS) in the Australian perinatal policy landscape and provide a foundation for future research in women whose scores indicate distress, defined as an EPDS score of 10 to 12. We suggest that despite scoring below 13, the threshold score for further specialised mental health assessment or intervention in the Australian setting, they are a logical group to whom specific attention should be paid and evidence-based population level preventative approaches applied. The emphasis of this article will be the antenatal period, as we wish to focus on the earliest time-point at which pregnant women engage with the universal perinatal care system, and when preventative efforts are likely to be most efficacious, though prevention strategies for postnatal depression will also be discussed.

### History of the Edinburgh Postnatal Depression Scale

The EPDS was introduced in 1987 [17]. When originally conceived, this 10-item self-report questionnaire was designed for use in women in the postnatal period, for the purposes of clinical practice and research. Previously used depression scales in the perinatal context, such as the Beck Depression Inventory (BDI) [18] or the Anxiety and Depression Questionnaire (SAD) [19], struggled to differentiate symptoms of depression from expected physiological experiences of pregnancy or the puerperium prompting development of the EPDS [20]. Subsequent studies indicated that the scale was also valid and reliable when used antenatally [21], although higher thresholds for clinical concern during pregnancy were suggested, prompting Cox and colleagues to recommend a change in nomenclature to the Edinburgh *Perinatal* Depression Scale [20]. The EPDS is the most widely used screening tool in both the pre- and postnatal period, though its evidentiary basis is more robust in the postnatal period [22].

### Choosing an appropriate cut-off score

Numerous validation studies, including the original work by Cox, Holden and Sagovsky [17] have demonstrated that a score of 13 or more strikes the optimal balance of sensitivity and specificity in classifying English-speaking women experiencing a major depressive episode postnatally [23, 24]. If the intention of screening is not to miss anyone who might be distressed and potentially vulnerable, arguments have also been put forward to increase sensitivity, and include the detection of minor depression by dropping the cut-off score to 10 or more [24, 25]. The extensive clinical use of this tool postnatally led to curiosity regarding its potential utility during pregnancy [26]. Antenatal validation studies subsequently conducted by Murray and Cox in 1990 [21] recommended a score of 15 or more for major depression and 13 or more for minor depression. The higher cut-off scores are hypothesised to be secondary to a lower prevalence of depression in the sampled population as well as the potential for misclassification of transient distress [27]. Further validation studies in other settings have confirmed the use of cut-offs of 13 or more during pregnancy [28], or a higher cut-off of 15 or more [29, 30]. Given the demonstration that transient heightened distress in pregnancy increases the risk of poor perinatal outcomes [31], and the demonstration that the test-retest reliability of the EPDS is high in a sample of the Australian population [32], one might argue that when this is identified, it should not be lightly dismissed.

In Cox, Holden and Sagovsky’s original 1987 validation study [17], using a cut-off score of 13 or more, the sensitivity of the EPDS was 86%, specificity of 78% and a positive predictive value of 73%. Cox recognised that failure to detect postpartum cases could be reduced to less than 10% by using a more conservative cut-off score of 10 or more which confirmed his earlier recommendation that this score be used for initial screening in the community setting [33]. The reliability of the EPDS in detecting meaningful changes in depression severity was also established in these early studies of its validity [20], broadening its utility in the primary prevention setting (through reapplication of the EPDS or as a justification for health promotion action) or as an outcome measure for targeted secondary prevention efforts (to assess effectiveness of an intervention and respond accordingly).

The acceptability of the EPDS to the woman, and for practitioners who are conducting the assessment, has implications for its accuracy and therefore utility as a screening test. Research in the Australian setting has confirmed a high level of acceptability of routine screening for both practitioners and pregnant or postpartum women [34, 35]. These findings have not necessarily been replicated in other settings, with Shakespeare et al. [36] describing 54% of surveyed women in their English sample as having found EPDS screening ‘unacceptable’. This study was limited by its small sample size of 39 women and stands in contrast to acceptability studies conducted across a range of cultural contexts [37]. Key ingredients identified as necessary for accurate completion of the EPDS were women having adequate prior preparation and information, interpersonal factors between mother and clinician, cultural appropriateness, confidence and competence of the clinician delivering the EPDS and skills in appropriately addressing disclosures [37]. We propose that these identified factors are particularly important for women who are experiencing sub-clinical symptomatology where symptoms are likely to be more challenging to elicit.

### Population level psychosocial assessment in Australia: embedding the EPDS

Australia-wide, antenatal psychosocial assessment including depression screening with the EPDS [38] is used to identify all levels of risk and current illness and respond with appropriate intervention. The EPDS was not, and is still not intended to be used without accompanying consultation. Decisions are not made on the basis of the scale score alone, especially if only one scoring occasion is reported [39]. Continuity of care and carer were emphasised as key ingredients of program delivery.

In practice, the accumulation of evidence and the desire for a pragmatic and sustainable approach have led to the use of antenatal EPDS scores of 13 or more as the point for further assessment and intervention. Again this assumes that all such women will need specialist assessment and/or referral. However, we continue to wonder whether lower scores, specifically 10 to 12, deserve to be better understood and more actively targeted through a population-based framework. A higher cut-off appears to assume that depressive illness is the only aspect to which attention should be paid, despite the similarly adverse functional outcomes associated with anxiety, stress and trauma, which are not specifically measured by this tool.

### EPDS improves detection of antenatal and postnatal depression

The introduction of the EPDS as a population-level screening measure has improved the rates of postnatal depression detection and offers superiority to unstructured health professional assessment [40, 41]. Prior to the Australia-wide roll-out of routine EPDS screening, Barnett and colleagues [42] applied the EPDS to 100 women admitted to an urban residential mothercraft program. Thirty-nine women scored above the set cut-off of 13 or more indicating probable major depression, with only one of these women having been identified as having postnatal depression prior to admission. Similarly, in a cohort of women studied by Hearn et al. [40], primary health care teams identified less than 50% of women scoring 12 or more on the EPDS as depressed. The primary health care team was superior in its detection rate compared with individual health practitioners, but still fell short of detecting the majority of women with probable depression.

The varying prevalence rates of perinatal depression between and within countries [7] prompt consideration of valid cut-off scores across different cultural groups. The EPDS has been translated into a number of languages, and while translational accuracy can be achieved, semantic and conceptual uniformity is more challenging to ensure.

Improved detection rates for postnatal depression led clinicians and policy makers to consider the routine use of the EPDS antenatally. Evans and colleagues [43] screened a large cohort of women in Avon, England between 1991 and 1992. Antenatal and postnatal EPDS scores were collected at 18 and 32 weeks gestation and again at 8 weeks and 8 months post-partum. Using a cut-off score of 13 and above, they showed higher mean scores during pregnancy compared with postnatal scores (9.2 and 8.1% of the sample at 8 weeks and 8 months postpartum respectively), with the highest risk period at 32 weeks gestation (13.6% of the sample). This finding was confirmed by Milgrom et al. [44] in the Australian context in a large cohort of women from across the country. While these studies are using surrogate measures for the presence of depression, the number alone is not the only triggering mechanism for referral or reassessment, but does demonstrate the value of a simple measure for population screening and improved detection.

### Psychosocial assessment including EPDS screening is therapeutic

As with any screening tool used in health service delivery, an appropriate intervention must be available and accessible, and the act of detection should not add to the burden of symptoms or the experience of stigmatisation [45]. In an early study, Malan et al. [46] demonstrated that depressive symptoms are in part relieved by the process of assessment, suggesting the importance of a warmly delivered, therapeutically-minded psychosocial interview which behaves at once as an assessment and therapeutic intervention. In Australia, Matthey and Ross-Hamid [47] examined 164 pregnant women who underwent EPDS screening as part of routine antenatal care. Regardless of which EPDS cut-off score was used (10 or more, 13 or 15) close to 50% of women improved to an acceptable or non ‘high-score’ 2 weeks later. This relation may be mediated by a validating environment in which the EPDS serves as an opportunity to discuss fears or concerns more broadly, or may indicate that the stress or distress was a temporary situation subsequently resolved; issues that would be clarified by the accompanying consultation [20]. Wickberg and Hwang [48] evaluated whether knowledge of a woman’s antenatal EPDS score affected midwifery management of depressive symptoms. This study showed that there was no difference in the rates of visits to the midwife, doctor or referrals to specialised mental health care practitioners, but that those women who were in the care of a midwife who was aware of their score at 25 weeks gestation had significantly improved scores on EPDS reapplication at week 36. These findings should relieve those at the coal-face of psychiatric or primary-care service delivery – that a one-off ‘high’ score alone will not lead to the ‘system’ being overwhelmed; instead, the screening environment is an opportunity for recognition, relationship-building and therapeutic counselling.

### Why should we focus on women scoring 10 to 12 on the EPDS antenatally?

Next, we examine the background and evidence for our recommendation that Australian guidelines should formalise care strategies for women who score 10 to 12 ante- or postnatally. Firstly we acknowledge that a woman who currently scores 10 to 12 is not necessarily dismissed by current guidelines [38]. Nevertheless we draw parallels between this group and their needs, and the environment of previous widespread under-detection. In a sea of conflicting priorities, time-poor and unsupported clinicians are incentivised to complete work rapidly and, while options may be available to this group of women if the clinician deems it appropriate, we argue that a formalisation of this framework will lead to capturing women who might have otherwise been missed. Again here we emphasise that decisions are not made on the score alone, but given the potential population reach of such universally delivered healthcare, this group also presents an opportunity for low-resource population-level prevention, early intervention and health promotion work. These population-level options could be delivered within existing models-of-care, such as midwifery group practice, or externally through a suite of low resource, personalised platforms, such as web apps.

### Anxiety is a core symptom of antenatal distress

Mainstream psychiatric taxonomy has long recognised the overlapping clinical features of depression and anxiety [49]. Clark and Watson [50] proposed a mixed depression-anxiety diagnosis in the early 1990s based on a tripartite model of illness, which suggested that general affective distress was the core dimension shared across diagnoses. Less inquiry has been afforded to the role of antenatal anxiety symptoms in the development of antenatal and postnatal depression, though it is a significant established risk factor [51]. Anxiety during pregnancy may present as a new or exacerbated condition. It may reflect pregnancy-specific anxiety arising from previous problematic outcomes or reproductive difficulties and may be particularly concerning for primiparous women, with first-time experiences of anticipating the pain of labour and childbirth, physical changes during pregnancy, miscarriage and financial strain [52]. Pregnancy-specific state or trait anxiety or distress has been demonstrated to be an independent risk factor for adverse obstetrical, neonatal and childhood development outcomes, including an increased likelihood of postpartum depression [53, 54], pre-term birth [55, 56], foetal hyperactivity [57] and later neurodevelopmental problems in the child [58, 59]. Compounding this association further is the increased likelihood of distress with subsequent births, in the context of previous traumatic or stressful pregnancy or birth experiences. This growing body of evidence has provided justification for assessing anxiety antenatally [60, 61], and providing therapeutic options, even if they are reinforcing simple measures ideally routinely delivered during maternity care, such as respectful, collaborative birth planning, reassurance or psychoeducation [62].

### The EPDS is multidimensional

The ensuing discussion around the biological and psychological origins of depression and anxiety has been absorbed into the perinatal mood disorder space, where clinicians have argued for the potential multi-dimensionality of the EPDS. Brouwers et al. [63] used principal component analysis to measure anxiety and depression sub-scales within the EPDS. This work demonstrated the existence of both depression and anxiety subscales, as well as non-specific questions that the authors have suggested may represent the overlapping affective distress common to these diagnoses and suggested by Clark and Watson’s tripartite model [50]. This finding has two important implications. Firstly, when the EPDS is being applied routinely during pregnancy and the postpartum period, it measures features of anxiety as much as depression. Secondly, when investigating associations between EPDS scores and risk factors or outcomes, subscale stratification may yield more specific and meaningful data on which to base policy recommendations.

### Evidence for action in the 10 to 12 space during the antenatal period

Next, our attention has turned to examining the potential effect of distress or sub-clinical symptoms including anxiety and stress symptoms, in the antenatal period, on maternal and infant outcomes. Hedegaard et al. [64] prospectively measured levels of psychological distress in a large cohort of Danish women and demonstrated a dose-response relationship between the presence of psychological distress at 30 weeks gestation and risk of preterm delivery.

The Avon Longitudinal Study of Parents and Children Study Team [65] demonstrated that antenatal depression was more common than postnatal depression [43], with literature supporting the finding that antenatal depression represents a significant risk factor for postnatal depression [66]. This finding is reflected internationally [67, 68], with the second and third trimesters of pregnancy presenting the highest risk [6]. This suggests that a proportion of women with postnatal depression are experiencing the continuation of untreated antenatal depression or that depressive symptom onset was within the antenatal period [26]. More recent literature including three systematic reviews [69–71], have demonstrated a range of deleterious physical and psychological effects on the developing foetus and neonates of mothers with antenatal depression. In a cohort of babies delivered in the public hospital system in Sydney, Australia, maternal depressive symptoms, defined as EPDS scores of 13 or more were associated with low birth weight and prematurity, though not breastfeeding initiation [16]. In a sample of African-American women, initial obstetric visit EPDS scores of 10 or more were correlated with increased risk of preterm birth, low birth weight and preeclampsia [72].

The consequences of an under- or untreated depression in the perinatal period threatens the attachment relationship of the mother-child dyad. Children of depressed mothers, when compared to children of non-depressed mothers, experience a range of neurocognitive, psychiatric and developmental adversity at higher rates. It is more likely that cognitive, intellectual and motor development is delayed, child temperament is more difficult and the quality of attachment between mother and child is affected resulting in poorer child emotional regulation and a higher rate of later behavioural problems [73–76]. These associations are compounded by an earlier manifestation of depressive illness, likely reflecting the higher sensitivity and responsiveness of an infant to parent-child interactions during this period [76]. Independent of perinatal depression, antenatal anxiety symptoms have been similarly demonstrated to be strongly associated with child behavioural and emotional problems at age 4 years in the Avon Longitudinal Study [58].

The prevention, earlier identification and treatment of perinatal anxiety and depression become the most important strategy in decreasing exposure of newborns to a major established risk factor for developmental adversity [73].

Understanding the trajectory and peak risk period of depression onset during pregnancy is central to the routine integration of screening instruments and the associated justification for treatment or increased monitoring. We suggest that antenatal depression has an early symptomatic stage, and that the EPDS has demonstrated value in identifying pregnant women in this stage when embedded in the full booking-in consultation which includes psychosocial assessment [77]. The mechanism via which the EPDS detects women in the precursor stages of distress could well be the presence of increased state anxiety, contributing to higher scores.

Women are reported to experience depression at least twice as commonly as men, with this demarcation commencing at puberty, and serving as the hypothetical basis that endocrinological fluctuations explain vulnerability to depression perinatally, menopausally and in the presence of hormonal contraception [78]. The role of hormonal changes and alterations in the hypothalamic-pituitary-axis are more convincing in the early postpartum period when oestrogen rises exponentially to 1000 times its usual levels, then decreases dramatically once the placenta has been delivered [79]. This has led to the hypothesis that a steroid-withdrawal response may account for postpartum blues and postpartum depression. The biological mechanisms causing depression antenatally have focused on the experience of maternal distress and its resultant effects on stress hormone production [80]. Understanding these biological mechanisms has important implications for determining the optimal time to screen women antenatally, and remains a high priority for biological researchers. Paternal depression during the perinatal period has also experienced increased research efforts with a reported prevalence meta-estimated at 8.4% (95% CI 7.2, 9.6) [81]. These comparable figures have led to recommendations that fathers also be screened for mood disturbance in the perinatal period, especially in the recognised high-risk circumstance of the mother experiencing depression [82].

### Risk-stratification properties of the Edinburgh postnatal depression scale

At some stage, it must be decided what percentage of distressed women may be missed in order to minimise false positive results. In the context of a self-report scale, the concept of sensitivity and specificity may not be as helpful. We argue that regardless of underlying pathophysiology, if a woman is scoring 10 or more the likelihood of subjective distress is high, and therefore we must be willing to accept a lower specificity in order to allow a comparably higher sensitivity. The potential harms of a false positive result may include stigmatisation or a sense of over-medicalisation of distressing feelings. Perhaps the solution then, lies in conceptualising the results of the EPDS as a spectrum. For pragmatic reasons, a cut-off measure is necessary, and behaves as a gatekeeper to resource-limited specialist services, though the decision to act or not must always rely on information obtained from the accompanying consultation. We argue that reliance on a binary outcome for further assessment or support services does not reflect the nature of mental health difficulties in the antenatal period, which is a time of instability and vulnerability. Instead, a more dynamic framework might recommend a low resource population-based intervention for women experiencing distress and reserving the more intensive resources for those with likely diagnosable illness. A model in which women scoring 10 to 12 are seen as displaying early symptoms (as in Wilson and Jungner’s model) [45], or sitting within a vulnerability or latency period where interventions may act therapeutically and preventatively. We suggest that aligning policy frameworks to include stepped-care options for women scoring 10 to 12 is a model which may in part address the current shortfall in providing interventions for women who score below a pragmatically, and not ethically, determined cut-off. Stepped-care models have become standard practice in the delivery of mental healthcare in Australia, and have made the benefits of functional interventions available to those who do not have diagnosable mental illness, but may have burdensome symptoms [83]. Despite the potential ability of the EPDS to stratify risk during pregnancy, integration of stepped-care models have not been trialled.

### Population-level interventions for women scoring 10 to 12 during perinatal assessment

Despite the availability of evidence-based treatments and pathways to care for depression, implementation gaps exist and a minority of women receive appropriate care [84]. We are particularly interested in recommending interventions that can be incorporated into current frameworks and service delivery models to address a significant proportion of women who are signalling distress.

Models of care for women with perinatal mental health disorder have to date used a tertiary prevention focus, that is, identification of individuals experiencing illness, then offering these individuals a service or treatment. We seek a population strategy, taking the form of primary or secondary prevention, as Rose [85] suggests, to alter the level and number of risk factors populations are exposed to. We suggest, as others have, that interventions delivered as part of routine care arrangements are more likely to be acceptable to the community, produce the least stigmatisation and attempt to address risk factors that exist for all women regardless of their age, socioeconomic status or cultural background [85, 86]. The perinatal period is highly complex with profound biological changes, psychological vulnerability and socio-occupational upheaval, so unsurprisingly protocols that focus on predictive tools alone do not consistently produce acceptable results [87]. Here, we focus on secondary prevention for women who score 10 to 12 on the EPDS, who are signalling distress, but who are currently underserved by guideline recommendations. Primary prevention models are not further explored, but may include interventions aimed at enhancing resilience, improving the couple relationship and practical planning for early parenting. At all levels, continuity of care and reliable social support remain key ingredients.

The breadth of trial research exploring preventative strategies for postnatal depression rarely deliver interventions during the antenatal period, and only one Australian trial used an EPDS score of 10 to 12 as inclusion criteria to assess the effectiveness of a sustained nurse home visitation program in preventing postnatal depression, among other maternal and infant outcomes [88]. This stands in contrast to the demonstration that application of a risk stratification procedure is more likely to produce greater intervention effects [89]. We review three interventions that warrant further consideration: couples-based interventions, telephone-based peer support and internet-delivered cognitive-behaviour therapy (iCBT).

### Couples-based interventions

Lack of partner support has been consistently demonstrated as a risk factor for both antenatal and postnatal depression in Australia and internationally [44, 67]. Interventions that target improved communication and understanding between partners seem a logical point for further review. Fisher and colleagues [86] conducted a before and after study where maternal and child health nurses invited first-time parents of infants at the universal first home visit to participate in a primary-care half-day psychoeducational group program focusing on infant behaviour management and adjustment tasks in the partner relationship. The intervention reflected the importance of postpartum anxiety in the pathogenesis of postpartum depression, but recognised that they are not readily distinguishable. The intervention was found to be protective against developing postnatal depression (OR 0.43, 95% CI 0.21, 0.89). This intervention is worthwhile for multiple reasons. Firstly, it has demonstrated its effectiveness as a low-resource preventative intervention. Secondly, and perhaps more importantly, perinatal depression, though not qualitatively different from depression at other points in the life cycle, does demonstrate a unique weight of risk factors, including increased reliance on and sensitivity within intimate partner relationships [86]. Brown and Harris’ [90] social theory of depression asserts that depression is initiated and driven by interpersonal factors, and given the significant socio-occupational and interpersonal changes during the antenatal and postnatal period, focus on this context is required if symptom remission is expected [91]. Indeed, this theory may also explain the phenomenon of postnatal depression in fathers. An unexpected benefit of this intervention may be to equalise the responsibilities and rewards of active parenthood for men.

### Telephone-based peer support

Dennis and colleagues [92] explored the potential effectiveness of telephone-based peer support in preventing postnatal depression in new mothers considered at “high-risk” of developing depression, as defined by an EPDS score of greater than nine, using a randomised controlled-design. The underpinning principle leading to the conception of this intervention was previous population-based studies that revealed women felt the cause of their postnatal depression was isolation and lack of support from other mothers [93, 94]. Peer-workers were women recruited by a convenience community sample in Ontario, Canada, who had reported a resolved history of postnatal depression. Peer-workers were provided 4 h of training. Women randomised to the intervention arm were matched to a peer-support mother according to area of residency and ethnicity if desired by the trial participant. Peer-mothers made a minimum of four contacts with the new mother then continued contact as agreed. Women were assessed at 12 and 24 weeks postpartum with the EPDS and a structured clinical interview. Fourteen percent of women in the intervention arm and 25% in the control arm had a score of greater than 12 at 12 weeks’ follow-up (relative risk reduction of 0.46, 95% CI 0.24, 0.62, *P* < 0.001). State-trait anxiety, measured by the Spielberger state-trait anxiety inventory [95], was also reduced in intervention compared with control mothers at 12 weeks follow-up, though fractionally failed to reach statistical significance (OR 1.4, 95% CI 0.99, 2.10, *P* = 0.055). These improvements were unsustained at 24 weeks follow-up. These results may have been secondary to contamination of results given women identified with depression at 12 weeks were referred for treatment and ceased to receive ongoing peer-support. A number needed-to-treat of eight women was calculated, to prevent one case of postnatal depression, and 80% of women reported satisfaction with the experience and would endorse the program to friends. There were some acknowledged methodological limitations in this paper however these were likely to be distributed equally across both study arms, so should allow the results of this unique study to be accepted.

### Internet-delivered intervention

Internet-based intervention holds promise for addressing barriers to detection and treatment for common mental disorders in the ante- and postnatal period. Though online or app-based treatment modalities have demonstrated benefit for a range of mental disorders [96], research to understand the legitimacy of these techniques in perinatal populations lag behind. Loughnan and colleagues conducted two randomised controlled trials of an internet-delivered brief and unguided internet cognitive behaviour therapy (iCBT) for the treatment of depression and anxiety symptoms in the antenatal period, ‘MUMentum pregnancy’ [97] and ‘MUMentum postnatal’ in the postnatal period [98]. Women were eligible if they demonstrated at least moderate depressive or anxiety symptoms, as measured by the Patient Health Questionnaire-9 and Generalised Anxiety Disorder-7 scales, respectively.

The MUMentum pregnancy trial [97] followed up pregnant participants and demonstrated significant reductions for both psychological distress and anxiety symptoms after application of three sessions of self-guided iCBT. These benefits were not sustained at 4 weeks follow-up. Limitations in study design included generalising results to a diverse group of women as the majority of participants had low levels of social stress (such as poor education, lack of partner support, unemployment). The MUMentum postnatal trial [98] produced significant improvements in anxiety, depression and distress symptoms at post-treatment review, with these benefits sustained at 4-weeks follow-up. Limitations in its trial design relate to the applicability of these results to women who may be subthreshold for a diagnosis of major depression or anxiety, women who are culturally and linguistically diverse and women with current substance use.

Future research could focus on testing the suitability and efficacy of web-delivered applications to women who are distressed, though subthreshold for diagnosis, across the trajectory of the perinatal period. Current population-delivered screening procedures in the perinatal period could support stratifying women for inclusion into this research space. Web-based applications have multiple benefits as an adjunct to current service delivery including continuous, confidential and accessible assessment at multiple time points and provision of a rationalised stepped-care approach.

### Conclusion and future directions

The burden of perinatal depression, the potentially irreversible effects of undetected illness on child life-course development and the potential for rare but catastrophic maternal and foetal consequences suggest that stepped-care systems with a preventive focus are justified and necessary. Furthermore, though effective treatments are available for postnatal depression, the complex and rapidly shifting context in which it is experienced results in implementation gaps and missed opportunities. Furthermore, a significant proportion of women who score below the antenatal and postnatal EPDS threshold value for optimal sensitivity and specificity for a clinical diagnosis of depression (13 or more), experience meaningful distress which is likely to exert similar emotional and physiological stressors on both the mother and infant as in the case of women scoring above the threshold.

The EPDS is already delivered nation-wide as part of a comprehensive, population-based psychosocial assessment in the public system. We suggest that we have the current capacity to identify women who may not be depressed, but who are signalling vulnerability, distress or dysphoria. Interventions for this group of women need not be labour-intensive, rely on specialist psychiatric services or focus on specific treatment modalities. Ideally, a suite of evidence-based, interventions should be available to clinicians and guided by a stepped-care approach. In its simplest, but not least powerful form, this might involve ensuring a newcomer to the area is provided with high-quality psychoeducational material, an antenatal support group or is aware of her options in terms of an appropriate primary care physician. For women who are signalling distress during either the psychosocial assessment, or scoring 10 to 12 on EPDS screening, it may involve offering access to a reputable phone or web application for further assessment and intervention. In all instances, a woman should be offered continuity of care and followed-up with further assessment as appropriate.

A similar stepped-care approach could be tailored to the postnatal period, with content modified to the specific changes and challenges experienced in new parenthood. Universally delivered postnatal EPDS screening could be utilised to stratify women into risk groups, with distressed women who are identified in the score range of 10 to 12 offered proactive intervention, rather than reassessment at a later time point. An additional benefit of using a dynamic web or phone-based intervention includes in-built ongoing assessment that can facilitate new parents seeking support sooner in the event of symptom deterioration.

Research on the trajectory and outcomes for women who are distressed in the perinatal period is gaining traction. The enthusiastic uptake of the EPDS as an easily-applied, readily acceptable, cross-culturally valid and brief instrument provides strong justification for exploiting its capacity more wholly. Cut-off figures are useful for determining predictive values but in time-poor, unsupported practice may have the unintended consequence of discounting genuine distress that is categorised as ‘improbable’ for depression. Though this cut-off is not arbitrary, the controlled conditions in which validation studies have taken place are unlikely to reflect the multiple factors at play when the EPDS is being applied in the field, and suggests that intervening at relatively lower scores appears sensible. While there are trials that examine interventions to prevent antenatal and postnatal distress in well populations, less research has focused on preventative interventions for women who are distressed, though perhaps not depressed during pregnancy and postpartum, despite the availability and robustness of routine screening architecture. Lastly, anxiety symptoms are less likely to be recognised by an EPDS cut-off score of 13 or more, so any approaches targeting distressed women should attempt to clarify and address the driver of distress.

The design and implementation of the NSW SafeStart guidelines were intended to facilitate psychosocial assessment and depression screening in the perinatal stages in consultation with the woman. A lack of pathway guidance and availability of interventions for women signalling distress but scoring less than 13 has emerged as a gap in program delivery. Since the relationship between professional and patient is fundamental, similarly a failure to act on the acknowledged wish of women and midwives for appropriate services that provide continuity of care and carer, is problematic. By ensuring adequate professional training and integrating a suite of available interventions endorsed within current guidelines for women scoring 10 to 12, a cultural shift in how the EPDS is utilised and maximised may follow. We also recognise that the current psychosocial assessment appropriately identifies trauma-related conditions, but does not offer appropriate further trauma-related inquiry or a suite of targeted interventions. Future directions in research would consider further characterising the validity of interventions specifically for distressed women scoring 10 to 12 on the EPDS, especially those measures that could be embedded within the currently delivered universal care system, and establish their preventative value.

## Data Availability

Not applicable.

## Abbreviations

ADQ: Anxiety and Depression Questionnaire
BDI: Beck Depression Inventory
EPDS: Edinburgh Perinatal Depression Scale
NSW: New South Wales
